# Improving surgical excellence: first experience of a video‐based intervention in outpatients

**DOI:** 10.1111/ans.18012

**Published:** 2022-09-02

**Authors:** Nelson Granchi, Jessica Reid, Katarina Foley, Amanda Le Couteur, Suzanne Edwards, Rebecca Feo, Markus Trochsler, Martin Bruening, Guy Maddern

**Affiliations:** ^1^ Discipline of Surgery The University of Adelaide, The Queen Elizabeth Hospital Woodville South South Australia Australia; ^2^ School of Psychology The University of Adelaide Adelaide South Australia Australia; ^3^ Adelaide Health Technology Assessment, School of Public Health The University of Adelaide Adelaide South Australia Australia; ^4^ College of Nursing and Health Sciences Flinders University Bedford Park South Australia Australia

**Keywords:** coaching, intervention, non‐technical skills, quality improvement, surgery

## Abstract

**Background:**

There are limited opportunities for surgeons to engage in active learning programs once they achieve Fellowship, especially for non‐technical skills such as communication. This study aims to address this gap by evaluating a peer‐based coaching program in non‐technical skill using video‐recorded patient consultations from a routine outpatient clinic.

**Methods:**

Standard outpatient consultations between consultant surgeons and patients were video recorded. The surgeon viewed the videos with a peer‐coach (senior surgeon) who helped identify areas of strength and areas for improvement. To test the effect of the coaching session, outpatient consultations were recorded roughly 1 month later. Pre and post‐coaching videos were assessed using the Maastricht History‐Taking and Advice Scoring – Global Rating List (MAAS), a common tool for evaluating non‐technical skills in clinicians.

**Results:**

A total of 12 surgeons consented to participate. Coaching significantly improved MAAS scores (mean difference = −0.61; 95% CI (−0.88, −0.33); *P* < 0.0001). Surgeons were generally positive about the experience. All found the method of learning suitable, and most thought the process improved their skills. Most thought that coaching would improve patient outcomes and the majority thought they would participate in ongoing coaching as part of their employment.

**Conclusion:**

This supports the concept of surgical coaching as an effective tool to improve communication skills and the quality of surgical consultation. The next step is to expand beyond a voluntary cohort and link surgical coaching to improved patient outcomes.

## Abstract 2

We sought to understand if a peer‐based coaching program can better the non‐technical skills of surgeons in the outpatient clinic environment. This interventional pilot study assessed 12 surgeons and was conducted at The Queen Elizabeth Hospital (TQEH) in Adelaide, Australia. Our results support the concept that a video‐based peer‐coaching program can act as an effective tool to improve the communication skills of consultant surgeons and improve the quality of surgical consultation.

## Introduction

Surgical coaching of non‐technical skills is an emerging field. While professional coaching is an established practice in other high‐performance fields such as sport, music, business and aviation, its value for surgeons has only recently been recognised.^1^, ^2^ Coaching is a process that aims to refine and improve existing skills through collaborative analysis and constructive feedback. Peer coaching involves colleagues, typically of similar experience, participating in a supportive interaction where the coach provides constructive feedback and encourages self‐evaluation to achieve specific goals.^3^, ^4^

Surgical trainees are typically provided with structured learning programs throughout their training, but this largely ceases on being awarded their Fellowship.^1^, ^2^, ^5^ Continuing professional development (CPD) is an ongoing requirement for consultant surgeons, but programs are based around passive learning^6^ and are not consistent with adult learning principles.^7^ Despite evidence that didactic forms of CPD are less effective in modifying physician practice,^8^ the majority of current CPD for surgeons remains didactic in nature, consisting mostly of one‐off events without follow‐up.^9^ Given that coaching allows participants to take an active role in their learning and identify strengths as well as areas for improvement, as per adult learning theory, peer‐based coaching has been proposed as a valuable form of CPD for practicing surgeons.^9^, ^10^

The majority of studies on coaching for fully qualified surgeons have centred on operative performance.^5^, ^11^, ^12^ Studies that focus on coaching surgeons in non‐technical skills in the outpatient clinic environment are largely absent from the literature. Additionally, learning opportunities for consultant surgeons skew towards procedural skills despite the emphasis placed by the Royal Australasian College of Surgeons on non‐technical skills such as communication, situational awareness and decision making in their core competencies framework.^13^, ^14^ Although technical skills are central to a surgeon's practice, non‐technical skills are vital for successful patient interaction; as evidenced by surgeons being more than twice as likely to receive patient complaints than their physician colleagues, with higher rates of dissatisfaction pertaining to interpersonal skills, communication and professional ethics.^15^ The outpatient clinic is of particular importance as it is here that patients are informed about their condition and have the opportunity to seek clarification and make decisions about their healthcare. Nonetheless, there is limited published data on how to conduct effective surgical outpatient consultations.

This study aims to address this gap by evaluating a peer‐based coaching program in non‐technical skills for consultant surgeons based on video‐recorded patient consultations from a routine outpatient clinic.

## Methods

### Setting and participants

This interventional pilot study was conducted at The Queen Elizabeth Hospital (TQEH) in Adelaide, Australia, with approval by the Central Adelaide Local Health Network (CAHLN) Ethics Committee (reference number HREC/17/TQEH/284). TQEH is a 300‐bed tertiary centre, where roughly 18 000 surgical procedures are completed per year by ~100 surgeons. Surgeons were eligible to participate as a ‘coachee’ if they were employed as a surgical staff consultant, surgical visiting medical officer, or a surgical fellow (FRACS achieved or equivalent). Coachees were given an information sheet and gave written informed consent to participate. The purpose of the study was explained to all patients who were scheduled to see the enrolled consultant during the clinic session. Patients were allowed time to consider the study and provided with a consent form. If a patient did not consent, their consultation was not recorded. Four experienced surgeons were identified by the Investigators as suitable coaches, each had over 15 years' experience as a consultant surgeon and had worked in leadership positions within a tertiary setting. Each coach was assigned three coachees.

### Coaching intervention

Coachees met with their assigned coach to evaluate their self‐perceived consultation skills. During this meeting, coachees outlined their goals for the program. These discussions were kept confidential from the study investigators and will not be published. An experienced human‐factors psychologist and lead author coordinated a training meeting for coaches with opportunities to discuss effective communication, imparting feedback and goal‐setting strategies.

Coaches and coachees met in a private space and for at least 1 h per session. During the coaching session, coaches and coachees engaged in structured discussion and free‐form conversation. The coach led the discussion while encouraging self‐assessment by the coachee. To formalize the discussion and provide a learning direction, a ‘Performance summary and action plan’ was created by the coachee. Categories included exploration, emotions, information giving, summarisations, structuring and empathy.

### Data collection

Coachees treated surgical patients in their normal outpatient clinic sessions. Video and audio data were recorded for these sessions using SIM Capture technology. Two small cameras (roughly the size of a mobile phone) were set up in each clinic room, one facing the consultant and the other facing the patient. The cameras were visible but were placed in unobtrusive locations, away from participants' eyeline. The cameras only recorded interactions between the consultant and the patient while they were seated. Physical examinations or requirements to undress occurred off‐camera in a separate room. Following clinic, coaches reviewed the collected data in private. After this, coaches and coachees met in person and discussed strengths, weaknesses and areas for improvement using the video as an objective tool. This overall process was repeated three times.

### Video selection

Using the SPSS random number generator, two videos were selected from each recorded clinic session. This resulted in six videos for each coachee: two prior to any coaching, two after one session of coaching, two after two sessions of coaching, a total of 72 videos for the cohort. The videos were provided to the relevant coaches for review prior to coaching sessions. At the end of the invention, these selected videos were provided to the Assessors.

### Outcomes and assessment

The Maastricht History‐Taking and Advice Scoring (MAAS)‐Global Rating List^16^ was employed as an assessment tool to measure the coachees' progress over the duration of the study. It was selected as the most suitable test to rate clinician‐patient interactions, because of its reliability and validation. MAAS‐Global combines 129 behavioural items and covers the entirety of a medical consultation. The areas scored by MAAS‐Global include: entry, overall orientation, exploration of the reasons for encounter, diagnostic plan, history taking, evaluation and giving information, management plan and evaluation of the consultation.

Four independent assessors reviewed the video recordings and evaluated the coachees non‐technical skills. Two were consultant surgeons with training in non‐technical and consulting skills and two were human‐factor psychologists with experience in medical quality improvement. Each assessor scored all 72 videos using MAAS. Assessors did not act as coaches during the intervention. Videos were blinded prior to review and assigned in a random order such that assessors were unaware of how many coaching sessions had occurred (0, 1 or 2).

Coachees assessed themselves using MAAS scores pre and post‐intervention. Following completion of the intervention, coachees were asked to assess the program using a Likert‐style questionnaire and free‐flowing feedback.

### Statistical analysis

The statistical software used was SAS 9.4 (SAS Institute Inc., Cary, NC, USA). To investigate the efficacy of a peer‐based coaching intervention for improvement of surgical outpatient consultations, a linear mixed‐effects model was performed. Initially, an interaction model was performed, however, as the interaction was not significant (*P*‐value = 0.3), the main effects model was used. The outcome was coach‐assessed MAAS score, and predictors were coaching session (baseline, 1 and 2) and video (1 and 2). A compound symmetry covariance structure was used to adjust for repeated measurements over time and a random effect for clustering on ID (two videos) was also included. Assumptions of a linear regression were found to be upheld by inspection of scatter plots and histograms of residuals and predicted values.

To investigate accuracy of self‐assessment of consulting skills (pre and post‐intervention), a linear mixed‐effects model was performed. The outcome was MAAS score, and the predictor was type (pre‐self‐reported, post‐self‐reported and at coaching 1/video 1). A variance components covariance structure was used to adjust for the random effect of clustering on ID (three types of assessment for each surgeon). Assumptions of a linear regression were found to be upheld by inspection of scatter plots and histograms of residuals and predicted values.

## Results

Twelve surgeons self‐nominated to participate in the program (September 2018–February 2019): 10 general surgeons, one urologist and one orthopaedic surgeon. To ensure confidentiality, limited demographic data were collected. All surgeons were consultants or fellows. There was one female surgeon. A total of 182 patients consented to participate.

Each coachee was able to complete the full study protocol of three recorded clinic sessions, with each clinic session followed by a coaching discussion. The mean time between the first clinic session and the second was 43 days (28–70 days), the mean time between the second recorded clinic session and the third was 37 days (21–68 days).

**Fig. 1:**
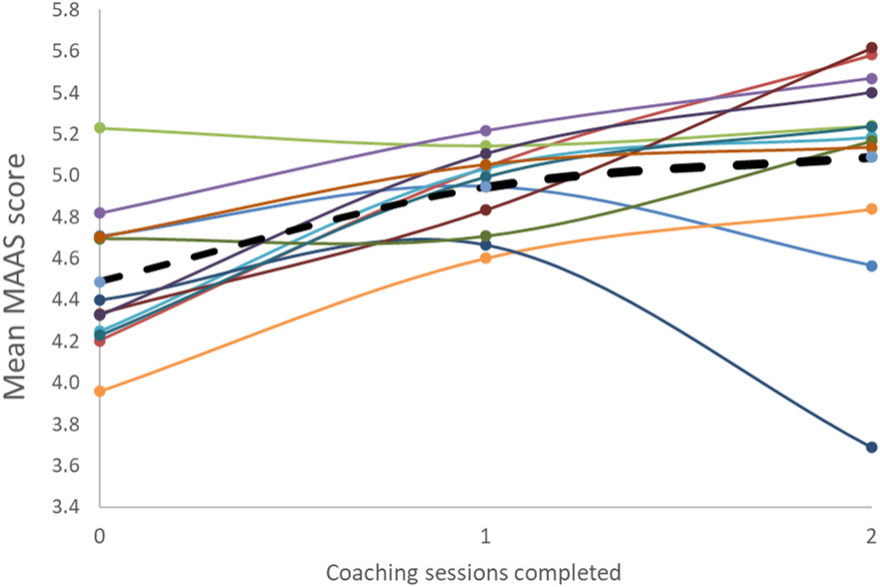
Most individual mean Maastricht History‐Taking and Advice Scoring (MAAS) scores increased from baseline after the first, and then second coaching sessions (solid lines). The overall mean score is shown by the black dotted line.

Over the course of the intervention, individual MAAS‐Global scores increased in 10 coachees, remained the same for one and decreased for one (Fig. 1). It was not possible to test whether individual coachees significantly improved after coaching due to insufficient power in the model. Linear regressions showed *p*‐values for all individual doctors to be >0.05.

However, the group as a whole demonstrated significant improvement after coaching sessions. A linear mixed‐effects model showed a significant positive association between mean MAAS‐Global scores and coaching, controlling for video and adjusting for repeated measurements over time (global *P*‐value = 0.0002). Mean scores improved from baseline versus post‐coaching session 1 (mean difference = −0.46; 95% CI: −0.74, −0.18; *P* = 0.0017) and baseline versus coaching session 2 (mean difference = −0.61; 95% CI: −0.88, −0.33; *P* < 0.0001). There was no significant difference in mean MAAS between coaching sessions 1 and 2.

Coachees assessed their own skills at baseline and post‐intervention and perceived an improvement in MAAS scores: 4.05 versus 4.71 (mean difference = 0.66; 95% CI: 0.25, 1.07; *P* = 0.0026). Assessor‐assessed MAAS scores were significantly greater than baseline self‐assessment: 4.47 versus 4.05 (mean difference = 0.41; 95% CI: 0.00, 0.83; *P* = 0.048), but not post‐intervention self‐assessment (*P* = 0.234). As mean baseline self‐assessed scores were lower than assessor scores, it may suggest that surgeons were tougher on themselves than the assessors.

Coachees were generally positive about the experience. All found the method of learning suitable, and 11/12 thought the process improved their skills (one unsure). Nine coachees reported that the coaching would improve patient outcomes (three unsure) and nine said they would participate in ongoing coaching as part of their employment (three unsure). No one reported feeling judged by their coach, and all said their views on their own performance were acknowledged and discussed. No coachee thought that the presence of video cameras had negatively affected the consultation, although there were mixed views on stress levels (two agreed, four unsure, and six disagreed), and on whether their behaviour was altered (six agreed, two were unsure, and four disagreed). Only one coachee reported concerns about ramifications of a sub‐optimal performance captured as part of the study. In response to the statement ‘I did not think I had deficiencies in my consulting skills’, three agreed, four were unsure and five disagreed. There were mixed responses to whether coachees preferred to select their own coach, two agreed, three were unsure and seven disagreed.

## Discussion

In this study, we sought to develop a peer‐based coaching program focusing on surgeons' non‐technical skills in the outpatient environment. Whereas previous studies investigating video‐based coaching for surgeons^5^, ^11^, ^12^ have focused on coaching in the operative setting, we chose to examine surgical coaching for outpatient clinic surgeon‐patient interactions.

Our results demonstrate that the communication skills of consultant surgeons improved following a video‐based peer‐coaching program. A single coaching session sufficed to significantly improve mean MAAS scores. However, measuring communication skills solely via MAAS scores is not without limitations, as various contextual factors in the doctor‐patient interaction may not be captured by a binary scoring system.^17^

A key factor in the success of the program is the opportunity to review video‐footage of the outpatient consultation; this enables the coach to highlight areas for improvement to the coachee directly. It also allows the coachee to re‐examine their practice at some distance and to avoid erroneous recall. This is beneficial as evidence indicates that doctors are not always the best judges of their own skills.^18^ Poorer performing surgeons are more likely to overestimate their skills^18^ which, in turn, may result in them being less likely to enrol in a voluntary coaching program. For this small subset, it is vital that coaching pivots from an optional exercise to a mandatory requirement, similar to basic life support, so that everyone is engaged.

While the ultimate goal of a peer‐based coaching intervention for surgeons is to improve the quality of care delivered to patients, surgical coaching is in its infancy and there is a lack of robust data to demonstrate that coaching improves patient care, including in this study where the improvement has been measured by an academic tool, not patient outcomes. Greenberg *et al*.^18^ sought to assess the impact of coaching on patient care and found that while operative times improved following a surgical coaching program, there was no significant improvement in risk‐adjusted outcomes. We are hopeful that our pilot study will pave the way for larger‐scale coaching interventions where effects on patient outcomes can be measured. Moreover, despite the success of coaching in other professions, the surgical discipline is yet to be convinced of the benefits for qualified surgeons. A recent survey conducted by this research group found that although surgeons generally support the idea of peer‐based coaching and acknowledge its potential as a form of CPD, they wish to see more evidence of its effectiveness.^19^ This could be achieved by highly powered randomized controlled trials.

In this study, all coachee surgeons completed the coaching program in the set timeframe, which is promising for the expansion of the program to a wider cohort, including other institutions. Despite this, many participants commented via their feedback that it was difficult finding time to schedule face‐to‐face coaching sessions. This issue has also been identified in other surgical coaching studies,^11^, ^12^ highlighting the need for hospital administrators to ensure time is quarantined for coaching sessions and for Colleges to replace passive learning CPD with coaching. A further solution, particularly in the COVID‐19 era, is for coaching sessions to be held virtually.

Further work is required on who can and should be a coach, as it is important that outdated practices or poor habits are not passed on. The coaches in our study were invited to participate by the Investigators as they regularly demonstrated behaviours in line with surgical excellence. This selection was similar to other studies where coaches were peer‐nominated.^20^ Additionally, studies have provided coaches with a similar level of training such as the Wisconsin Surgical Coaching Program and the Michigan Bariatric Surgery Collaborative where coaches received a half day of training on peer coaching roles and expectations.^20^

This is the first study centred on coaching surgeons' non‐technical skills in the outpatient environment with the goal of optimizing the surgeon‐patient interaction. It is important to note that we are not seeking a perfect, standardized outpatient consultation. The aim of coaching is to enable surgeons to refine and improve their non‐technical skills so that patient experiences of consultations are constructive, valuable and empowering.

## Conflict of interest

None declared.

## Author contributions

**Nelson Granchi:** Investigation; project administration; visualization; writing – original draft. **Jessica Reid:** Conceptualization; data curation; methodology; writing – review and editing. **Katarina Foley:** Writing – review and editing. **Amanda Le Couteur:** Data curation; methodology; writing – review and editing. **Suzanne Edwards:** Formal analysis; writing – review and editing. **Rebecca Feo:** Data curation; writing – review and editing. **Markus Trochsler:** Writing – review and editing. **Martin Bruening:** Writing – review and editing. **Guy Maddern:** Conceptualization; methodology; resources; writing – review and editing.

## Funding information

This study was funded by the Avant Foundation Grant to support initiatives that improve quality, safety and professionalism in practice.
